# Supplementary material for: Time-series transcriptome analysis identified differentially expressed genes in broiler chicken infected with mixed Eimeria species
Source: Front Genet. 2022 Aug 8;13:886781. doi: 10.3389/fgene.2022.886781 (PMC9393255; doi:10.3389/fgene.2022.886781)
Supplement: Supplementary file 2 [file DataSheet1.ZIP › 7dpi_KEGG.Gsea.1625115961950/KEGG_GLUTATHIONE_METABOLISM.html]

Details for gene set KEGG\_GLUTATHIONE\_METABOLISM[GSEA]

|  || Dataset | TMM\_7dpi\_gct\_format\_7dpi\_gct\_format.Class\_4dpi.cls #PC\_versus\_NC.Class\_4dpi.cls #PC\_versus\_NC\_repos |
| Phenotype | Class\_4dpi.cls#PC\_versus\_NC\_repos |
| Upregulated in class | 1 |
| GeneSet | KEGG\_GLUTATHIONE\_METABOLISM |
| Enrichment Score (ES) | 0.542652 |
| Normalized Enrichment Score (NES) | 2.2236364 |
| Nominal p-value | 0.0 |
| FDR q-value | 8.809342E-4 |
| FWER p-Value | 0.0034 |
Table: GSEA Results Summary

  

Fig 1: Enrichment plot: KEGG\_GLUTATHIONE\_METABOLISM      
 Profile of the Running ES Score & Positions of GeneSet Members on the Rank Ordered List

  

| SYMBOL | TITLE | RANK IN GENE LIST | RANK METRIC SCORE | RUNNING ES | CORE ENRICHMENT || 1 | GSTA3 | na | 22 | 1.645 | 0.0921 | Yes |
| 2 | MGST1 | na | 67 | 1.378 | 0.1671 | Yes |
| 3 | MGST3 | na | 120 | 1.205 | 0.2316 | Yes |
| 4 | GSTA2 | na | 139 | 1.157 | 0.2961 | Yes |
| 5 | GSTZ1 | na | 161 | 1.120 | 0.3583 | Yes |
| 6 | GSTT1 | na | 164 | 1.112 | 0.4217 | Yes |
| 7 | GPX1 | na | 190 | 1.058 | 0.4800 | Yes |
| 8 | SMS | na | 495 | 0.746 | 0.4972 | Yes |
| 9 | PGD | na | 604 | 0.684 | 0.5273 | Yes |
| 10 | GPX4 | na | 881 | 0.561 | 0.5363 | Yes |
| 11 | GSTO2 | na | 1249 | 0.441 | 0.5309 | Yes |
| 12 | GCLM | na | 1388 | 0.408 | 0.5427 | Yes |
| 13 | GSTK1 | na | 1770 | 0.314 | 0.5288 | No |
| 14 | GSTA4 | na | 1872 | 0.294 | 0.5371 | No |
| 15 | GPX7 | na | 3120 | 0.112 | 0.4394 | No |
| 16 | ODC1 | na | 3147 | 0.107 | 0.4434 | No |
| 17 | IDH2 | na | 3503 | 0.063 | 0.4173 | No |
| 18 | GPX3 | na | 3522 | 0.061 | 0.4193 | No |
| 19 | LAP3 | na | 3559 | 0.057 | 0.4196 | No |
| 20 | GSS | na | 3680 | 0.043 | 0.4120 | No |
| 21 | GSR | na | 4363 | -0.036 | 0.3571 | No |
| 22 | RRM2B | na | 5085 | -0.111 | 0.3033 | No |
| 23 | MGST2 | na | 5769 | -0.177 | 0.2563 | No |
| 24 | IDH1 | na | 5855 | -0.186 | 0.2599 | No |
| 25 | GSTM2 | na | 6008 | -0.202 | 0.2587 | No |
| 26 | TXNDC12 | na | 6413 | -0.244 | 0.2389 | No |
| 27 | RRM2 | na | 6728 | -0.278 | 0.2286 | No |
| 28 | GGT7 | na | 6952 | -0.302 | 0.2273 | No |
| 29 | SRM | na | 7272 | -0.337 | 0.2199 | No |
| 30 | RRM1 | na | 7510 | -0.362 | 0.2208 | No |
| 31 | GGCT | na | 8548 | -0.491 | 0.1622 | No |
| 32 | GCLC | na | 9266 | -0.593 | 0.1362 | No |
| 33 | ANPEP | na | 10055 | -0.720 | 0.1116 | No |
| 34 | GGT5 | na | 10882 | -0.903 | 0.0942 | No |
Table: GSEA details [plain text format]

  

Fig 2: KEGG\_GLUTATHIONE\_METABOLISM      
 Blue-Pink O' Gram in the Space of the Analyzed GeneSet

  

Fig 3: KEGG\_GLUTATHIONE\_METABOLISM: Random ES distribution      
 Gene set null distribution of ES for **KEGG\_GLUTATHIONE\_METABOLISM**
